# The random noise modulations on the nonlinear Chiral Schrödinger structures

**DOI:** 10.1371/journal.pone.0324833

**Published:** 2025-09-24

**Authors:** Hadil Alhazmi, Sanaa A. Bajri, E.K. El-Shewy, Mahmoud A.E. Abdelrahman

**Affiliations:** 1 Department of Mathematical Sciences, College of Science, Princess Nourah bint Abdulrahman University, Riyadh, Saudi Arabia; 2 Theoretical Physics Group, Faculty of Science, Mansoura University, Mansoura, Egypt; 3 Department of Physics, College of Science, Taibah University, Al-Madinah Al-Munawarah, Saudi Arabia; 4 Department of Mathematics, College of Science, Taibah University, Al-Madinah Al-Munawarah, Saudi Arabia; 5 Department of Mathematics, Faculty of Science, Mansoura University, Mansoura, Egypt; PLOS: Public Library of Science, UNITED KINGDOM OF GREAT BRITAIN AND NORTHERN IRELAND

## Abstract

In this paper, we consider the Chiral nonlinear Schödinger equation (CNLSE), where the multiplicative noises term varies arbitrarily over time. This equation defines several edge states of Hall effect characteristics in quantum physics applications. We apply the sine-Gordon expansion method to produce some new stochastic solutions for the CNLSE. Some solitary and dissipative solutions were obtained in the form of rational, envelope and shock structures. We demonstrate how the multiplicative noise and model parameters affects the way the solutions behave. We provide some configurations for the both deterministic and stochastic solutions to illustrate their behaviour. It is known that noise dominates envelope growing, damping, and all wave propagation. As it is achieved, the results presented here are crucial to the development of quantum physics. The proposed methodology can be developed to solve more complex problems in applied science.

## 1 Introduction

Mathematical simulation for several critical real-world problems often leads to differential equations and other problems; key mathematical physics functions, as well as their extensions and generalisations, are covered [1–6]. The best known of them occupy a place in the family of digital or explanatory plans, taking into account the method of the multimodal learning paradigm [7], multimodal hybrid parallel method [8], neural architecture technique [9] and improved BPNN approach [9]. The abilities of nonlinear systems that accurately express the dynamics of a wide range of events in science and nature are attracting a lot of attention [11–14].

Solitons are used to explain the structure of certain particular solitary waves in the nonlinear stochastic partial differential equations (NSPDEs). Moreover, there is great interest in the study of nonlinear wave dynamics in various physical contexts, which reflects various phenomena related to the properties of solitons [15,16]. These equations have recently attracted the attention of numerous scientists due to their success in simulating a variety of physical phenomena, including optical fibers, plasma physics, superfluid, HIV internal virus dynamics, chemical kinematics, astrophysical dynamics, electro magnetic wave propagation and other more [17–21]. As a result, it is critical to examine the dynamics of the solutions of the NSPDEs. Numerous researchers have employed NPDEs to create voyaging wave patterns using a variety of methods [22,23,27]. Recent research has focused on finding nonlinear model equations and problems, which has been extended to cases involving network traffic detection, neural architecture, pulse load, etc [28–30].

A stochastic process is an observation that takes place at a specific time and results in a random variable. The theory of stochastic processes was established mathematically around 1950. Since then, stochastic processes have developed into a standard tool for mathematicians, physicists, and engineers. The Wiener (Brownian motion) process is a prominent stochastic process that is both a Markov and a martingale process [31]. It’s been utilised in engineering, finance, physics, etc. This procedure is critical in the generation of NSPDEs.

The Schödinger equation represents a study of energy dynamics in many applied systems, such as medical physics applications, such as the movement of blood in veins and arteries and the effects on them of bacterial and physical diseases [32]. Studies on the effects of acoustic noise on human health have been conducted in recent decades. It irritates people and alters the risk of myocardial infarction, stroke, and arterial hypertension [33]. To lessen the negative impacts of noise on public health, theorists are attempting to use Schödinger equations to address the cardiovascular effects of exposure to ambient noise [34,35]. Solvable differential equations that assess the effect of mean blood pressure on the aorta wall and demonstrate the existence and uniqueness of its solution for homeostatic recoil and relaxation for minuscule aortic tissue are utilized to mathematically characterize the aortic dissection. Different types of Schödinger equations in quantum wells with arbitrary potentials can be dealt with using machine learning techniques. Two neural networks with different architectures are proposed and trained using a set of potentials, energies and wave functions that have been previously generated using classical finite element methods and other numerical methods. The equations are solved analytically, such as sine-Gordon expansion method by converting these equations to ordinary differential forms and finding their direct solution.

The nonlinear Schödinger’s equation (NLSE) represents wave propagation in mediums with both dispersive and nonlinear characteristics. This equation serves as the fundamental building block for characterising wave behaviours in a wide variety of important applications of applied research, like deep water, semiconductors, Bose-Einstein condensations, collapsing solar wind wave energies, bimolecular, etc [36–41]. As a model of energy transfer in a monolayer molecular aggregation with thermal fluctuations, the NLSE with multiplicative noise was introduced [23]. The authors proved the global existence and uniqueness of solutions to the stochastic logarithmic Schödinger equation induced by additive or multiplicative noise [24]. The authors consider the random effect of noise dispersion on the stochastic logarithmic Schödinger model arising in optical fibers [25]. The optimal control formulation for the stochastic nonlinear Schödinger equation on a finite graph was investigated [26]. The chiral nonlinear Schödinger equation (CNLSE) via multiplicative noise in the It ô sense is the governing model that will be examined in this research:

$$i\phi_{t}+\phi_{xx}-ia(\phi^{*}\phi_{x}-\phi \phi_{x}^{*})\phi +\sigma \phi \Upsilon_{t}=0,$$ (1)

ϕ(x,t) is a complex function of *x* & *t*, *α* is a nonlinear coupling constant and * indicates the complex conjugate. The noise $\Upsilon_{t}=\frac{d\Upsilon }{dt}$ is the time derivative of the Brownian motion Υ(t), whilst *σ* is the noise strength. The Brownian motion process is a stochastic process $\{\Upsilon (t){\}}_{t\geq 0}$ satisfies Υ(t), t≥0 are continuous functions of *t*, for *s* < *t*, Υ(s)−Υ(t) is independent of increments and Υ(t)−Υ(s) has a normal distribution with mean 0 and variance *t*–*s*. Both bright and dark soliton solutions were provided in an earlier investigation of the CNLSE [42]. Solitons for CNLSE are important in the setting of the quantum Hall effect, where chiral excitations are known to occur [43,44].

We produce some new stochastic solutions for the CNLSE via multiplicative noise in the Itô sense, using the sine-Gordon expansion approach. The key advantage of this technique is that it allows you to obtain different family solutions using free physical parameters. Numerous applied sciences, including nuclear physics, quantum mechanics, plasma physics, nonlinear optics, modern quantum Hall effect and nanotechnology, will greatly benefit from the solutions offered. Clarifying how the noise term affects the presented solutions is one of the more intriguing issues. We also demonstrate the nonlinear dynamical behaviour of some selected stochastic solutions.

The remainder of this paper’s framework is organised as follows. Sect 2 introduces a description of the proposed method. Sect 3 produces the stochastic solutions for the CNLSE through multiplicative noise via Itô sense. Sect 4 explains the obtained stochastic solution. Some conclusion are illustrated in Sec. 5.

## 2 Description of the method

We provide a condensed version of the sine-Gordon expansion method [45].

Consider the sine-Gordon equation [45]

$${𝒲}_{xx}-{𝒲}_{tt}=\rho^{2}\;\text{sin}(𝒲),$$ (2)

𝒲(x,t); *ρ* is a non-zero real number. Using wave transformation

𝒲(x,t)=Φ(ξ),ξ=x−vt. (3)

*v* is the wave speed, Eq (2) becomes

$$\Phi^{\prime \prime }=\frac{\rho^{2}}{1-v^{2}}\;\text{sin}(\Phi ).$$ (4)

Multiplying $\Phi^{\prime }$ on both sides of Eq (4) and integrating it gives [45]:

$${\left[{\left(\frac{\Phi }{2}\right)}^{\prime }\right]}^{2}=\frac{\rho^{2}}{1-v^{2}}{\text{sin}}^{2}(\frac{\Phi }{2})+R,$$ (5)

*R* is constant of integration. By putting *R* = 0, $\frac{\Phi }{2}=\psi (\xi )$ and $\frac{\rho^{2}}{1-v^{2}}=a^{2}$ into Eq (5) produces

$$\psi^{\prime }=a\;\text{sin}(\psi ).$$ (6)

Choosing *a* = 1, Eq (6) turns into

$$\psi^{\prime }=\text{sin}(\psi ).$$ (7)

The solution of Eq (7) is

$$\text{sin}(\psi )=\text{sin}(\psi (\xi ))=\frac{2Ke^{\xi }}{K^{2}\;e^{2\xi }+1}{|}_{K=1}=\text{sech}(\xi ).$$ (8)

$$\text{cos}(\psi )=\text{cos}(\psi (\xi ))=\frac{K^{2}e^{2\xi }-1}{K^{2}\;e^{2\xi }+1}{|}_{K=1}=\text{tanh}(\xi ),$$ (9)

where K≠0 is the integration constant. Now, consider the following NPDEs:

$$\Upsilon (𝒲,{𝒲}_{x},{𝒲}_{t},{𝒲}_{xx},{𝒲}_{xt},{𝒲}_{tt},...)=0.$$ (10)

Utilizing the wave transformation:


𝒲(x,t)=Θ(ξ),      ξ=x−vt,

Eq (10) converted to the following ODE:

H(Θ,Θ',Θ'',Θ''',...)=0. (11)

It is assumed that the solution Θ(ξ) of Eq (11) can be written as

$$\Theta (\xi )=\sum_{i=1}^{N}{\text{tanh}}^{i-1}(\xi )\left[\Lambda_{i}\;\text{sech}(\xi )+\Gamma_{i}\;\text{tanh}(\xi )\right]+\Gamma_{0}.$$ (12)

Considering Eqs (8) and (9), Eq (12) can be written as follows

$$\Theta (\psi )=\sum_{i=1}^{N}{\text{cos}}^{i-1}(\psi )\left[\Lambda_{i}\;\text{sin}(\psi )+\Gamma_{i}\;\text{cos}(\psi )\right]+\Gamma_{0}.$$ (13)

The homogeneous balancing concept is used to estimate the value of *N*. A system of algebraic equations is produced by substituting Eq (13) into Eq (11) and comparing the terms. Solving these equations yield the travelling wave solutions of Eq (10).

## 3 Mathematical analysis

Using the wave transformation:

$$\phi (t,x)=q(\xi )e^{i[cx+\omega t+\sigma \Upsilon (t)]},\;\xi =\beta (x+vt),$$ (14)

*q* is a real function, *c*, ω, *β* are nonzero constants. We have

$$\frac{d\phi }{dt}=(\beta vq^{\prime }+i\omega q+i\sigma q\Upsilon_{t})e^{i[cx+\omega t+\sigma \Upsilon (t)]},\;\frac{d\phi }{dx}=(\beta q^{\prime }+icq)e^{i[cx+\omega t+\sigma \Upsilon (t)]},\;\frac{d\phi^{*}}{dx}=(\beta q^{\prime }-icq)e^{-i[cx+\omega t+\sigma \Upsilon (t)]},\;\frac{d^{2}\phi }{dx^{2}}=(\beta^{2}q^{\prime \prime }+2i\beta cq^{\prime }-c^{2}q)e^{i[cx+\omega t+\sigma \Upsilon (t)]}.$$ (15)

Using (14) and (15) into Eq (1) gives

$$\beta^{2}q^{\prime \prime }+2acq^{3}-(\omega +c^{2})q=0.$$ (16)

from real part and v=−2c from imaginary part.

Balancing the highest order derivative and the highest nonlinear terms $q^{\prime \prime }$, *q*^3^ gives *N* = 1. As a result, the solution to Eq (16) is

$$q(\xi )=\Gamma_{0}+\Gamma_{1}\text{tanh}(\xi )+\Lambda_{1}\text{sech}(\xi ).$$ (17)

Superseding *q*, $q^{\prime \prime }$, and *q*^3^ into Eq (16) and setting the hyperbolic function coefficients to zero results in some algebraic equations that provide:

**Family I:**

The solution of Eq (16) is

$$q_{1}(x,t)=-\frac{\beta }{\sqrt{ca}}\;\text{sech}(\beta (x-2ct)).$$ (18)

As a result, the stochastic solution for Eq (1) is

$$\phi_{1}(x,t)=-\frac{\beta }{\sqrt{ca}}\;e^{i(cx+(\beta -c)(\beta +c)t+\sigma \Upsilon (t))}\;\text{sech}(\beta (x-2ct))\;,$$ (19)

c,a≠0.

**Family II:**

The solution of Eq (16) is

$$q_{2}(x,t)=-\frac{\sqrt{c^{2}+w}}{\sqrt{ca}}\;\text{sech}(\sqrt{c^{2}+w}(x-2ct)).$$ (20)

As a result, the stochastic solution for Eq (1) is

$$\phi_{2}(x,t)=-\frac{\sqrt{c^{2}+w}}{\sqrt{ca}}\;e^{i(cx+wt+\sigma \Upsilon (t))}\;\text{sech}(\sqrt{c^{2}+w}(x-2ct))\;,$$ (21)

c,a≠0 and *c*^2^ + *w*>0.

**Family III:**

The solution of Eq (16) is

$$q_{3}(x,t)=-\frac{\beta \sqrt[{4}]{\beta^{2}-w}}{\sqrt{a(\beta^{2}-w)}}\;\text{sech}(\beta (x-2\sqrt{c^{2}-w}t)).$$ (22)

As a result, the stochastic solution for Eq (1) is

$$\phi_{3}(x,t)=-\frac{\beta \sqrt[{4}]{\beta^{2}-w}}{\sqrt{a(\beta^{2}-w)}}\;e^{i(\sqrt{\beta^{2}-w}x+wt+\sigma \Upsilon (t))}\;\text{sech}(\beta (x-2\sqrt{c^{2}-w}t))]\;,$$ (23)

a≠0 and $\beta^{2}-w>0$.

## 4 Results and discussion

The model (1) was transformed via equation (14) to the differential equation (16). This equation introduces kinetic energy equation in the form

$$\frac{1}{2}q^{\prime }(\xi {)}^{2}=-\frac{1}{2}\frac{ac}{\beta^{2}}\;q(\xi {)}^{4}-\frac{1}{2}\frac{c^{2}}{\beta^{2}}q(\xi {)}^{2}+\frac{1}{2}\frac{\omega }{\beta^{2}}q(\xi {)}^{2}.$$ (24)

The exact solution for this model is

$$q(x,t)=\frac{2\left(c^{2}+\omega \right)e^{(x+vt)\sqrt{c^{2}+\omega }}}{\sqrt{ac\left(c^{2}+\omega \right)}\left(e^{2(x+vt)\sqrt{c^{2}+\omega }}+1\right)}.$$ (25)

Thus the corresponding stochastic form for Eq (1) is

$$\phi \left(t,x\right)=\frac{2\left(c^{2}+\omega \right)e^{(x+vt)\sqrt{c^{2}+\omega }}}{\sqrt{ac\left(c^{2}+\omega \right)}\left(e^{2(x+vt)\sqrt{c^{2}+\omega }}+1\right)}e^{i(cx+wt+\sigma \Upsilon (t))}.$$ (26)

In order to obtain more relevant solutions used in stochastic applications, the (1+1)-dimensional CNLSE has been solved using the sine-Gordon expansion technique due to certain constraints. Many researchers use the CNLSE to characterise physical occurrences in the quantum field of Fractional-Hall effect edge states [43,44,46]. A major portion of the laser’s operation is based on quantum physics. The behaviour of the supplied solutions, which can be solitons, blow ups, periodic, dissipative, and so on, is determined by the physical parameters in the CNLSE coefficients. For instance, at certain wave number, the wave’s profile picture’s attitude changes from compressive to rarefactive at critical spots, and from stable to unstable regions [47]. Since chiral excitations are known to arise in the quantum hall effect, the topic of chiral solitons solutions is crucial to its progress.

We investigated the chiral nonlinear Schrödinger equation induced by multiplicative noise via Brownian motion process. This process describes the haphazard movement of microscopic particles in gases and liquids. It is a basic procedure that can be applied to define more intricate stochastic procedures. It also forms the basis for the rigorous path integral formulation of quantum mechanics. By far the most important stochastic process is Brownian motion. The proposed chiral nonlinear Schrödinger equation was considered in most standard articles in the deterministic situation. Unlike our method, we consider these models in the stochastic situation. Actually, we developed some new stochastic solutions for the CNLSE in Itô sense via sine-Gordon expansion approach. To the best of our knowledge, the proposed outcomes in this paper have not been published.

**Fig 1:**
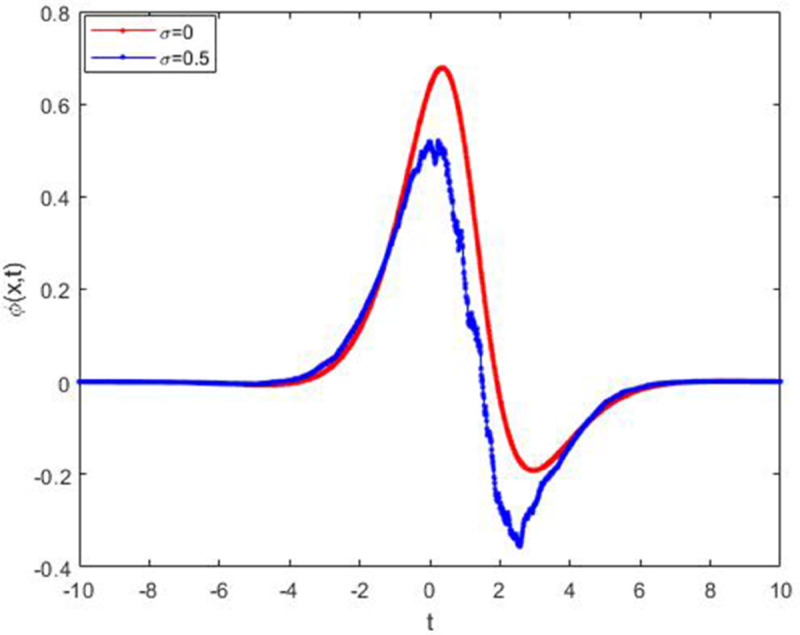
Influence of noise term on wave capture for solution (19).

**Fig 2:**
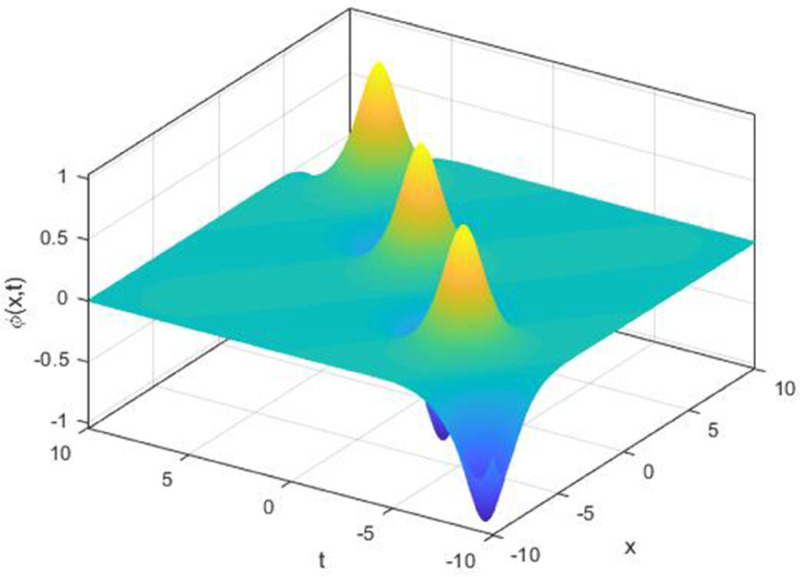
Profile graph of solution (19) for σ=0.

**Fig 3:**
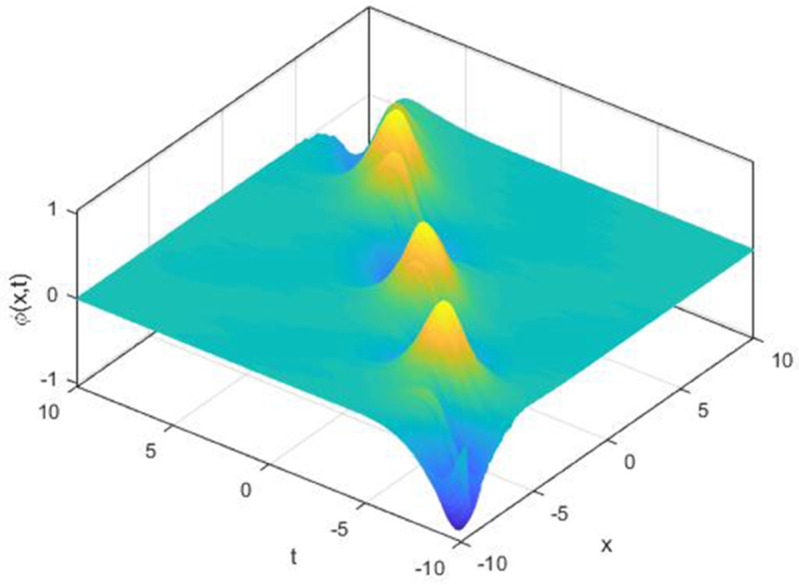
Profile graph of solution (19) for σ=3.

**Fig 4:**
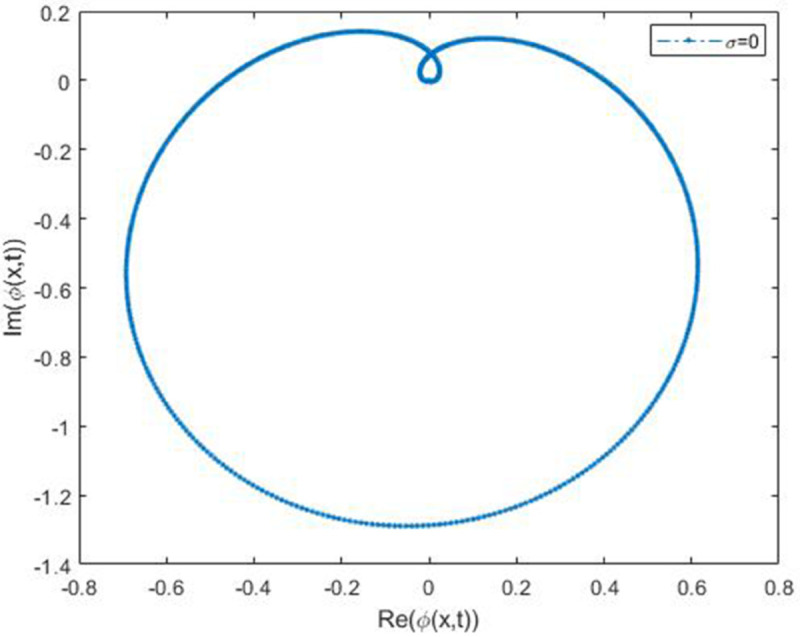
Profile graph of solution (21) for σ=0.

**Fig 5:**
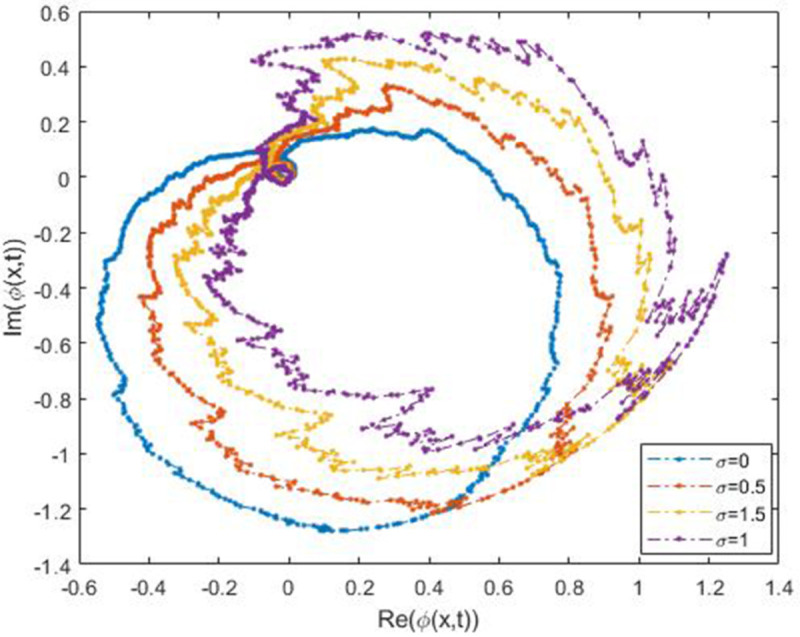
Trajectory of solution (21) via values of *σ.*

**Fig 6:**
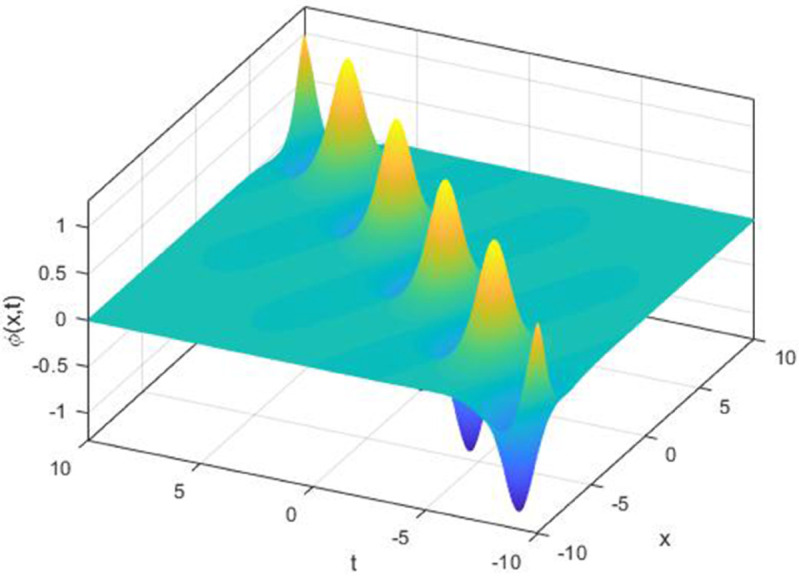
Profile graph of solution (21) for σ=0.

**Fig 7:**
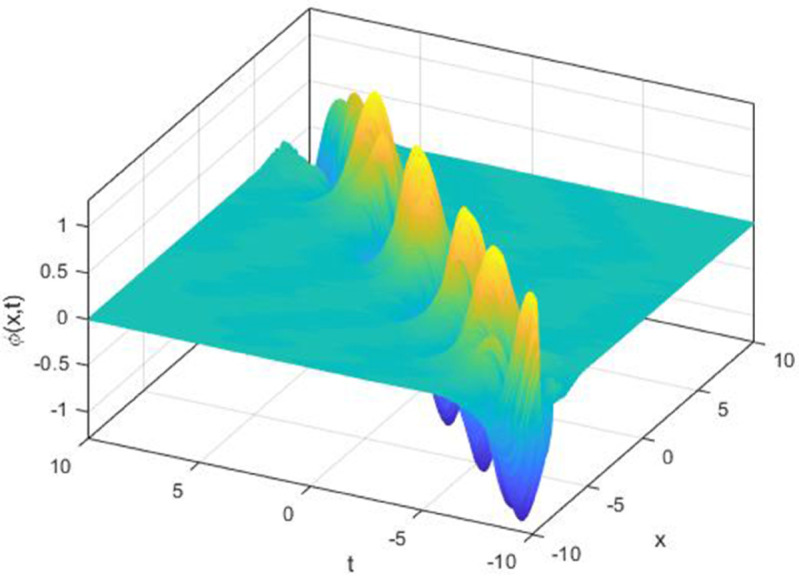
Profile graph of solution (21) for σ=5.

We explored the effect of noise parameter on the behaviour of the solitary waves. This parameter adjusts the wave characteristics, such as amplitude, frequency variations, and wave transformations [48,49]. Figs 1 - 7 exhibit the simulated behaviours of the stochastic solutions in deterministic case and stochastic cases. In Fig 1 the noise affects the wave amplitude with sharp distortion. The envelope soliton for solution (19) were plotted in Figs 2, 3 for σ=0,σ=3. It was noted that the marked reduction in envelope amplitude have been produced for σ=3. The trajectory of solution (21) have been depicted for deterministic and stochastic cases in Figs 4, 5. The stochastic noise effect on the stability of solution (21) has been shown in Fig 5. The stochastic noise effect on the frequency of envelope soliton for solution (21) have been reported in Figs 6, 7 for σ=0,σ=5.

**Fig 8:**
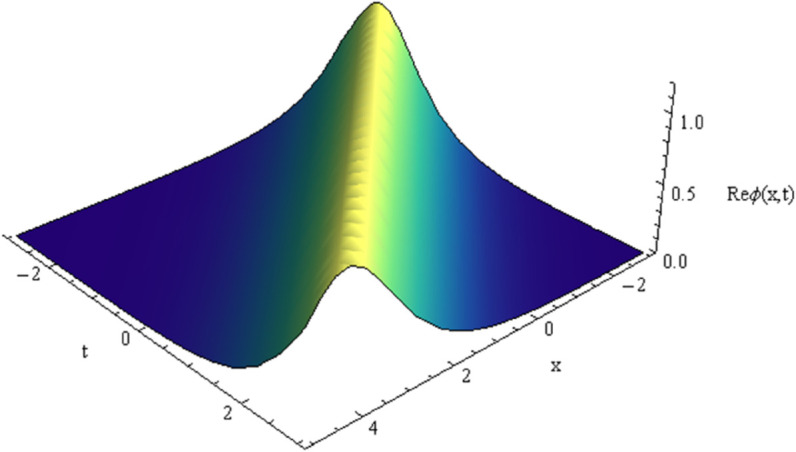
Plot of solution (26) with *x*,*t.*

**Fig 9:**
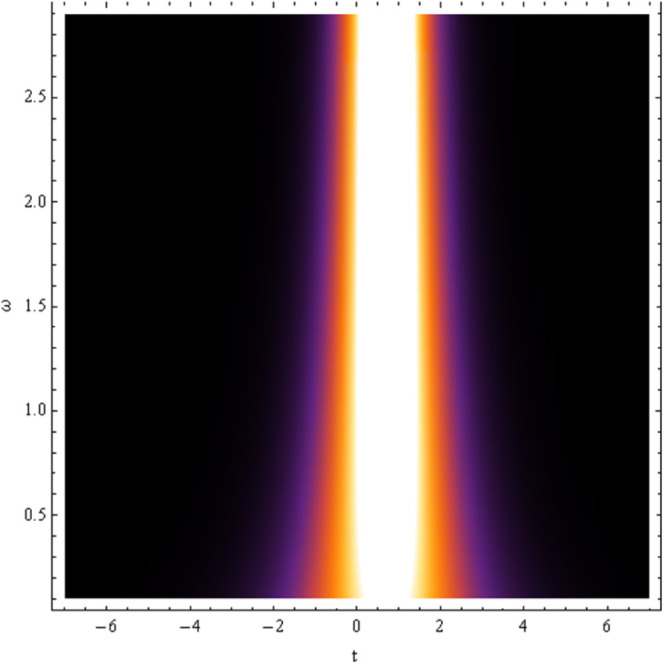
Plot of solution (26) with ω,t.

**Fig 10:**
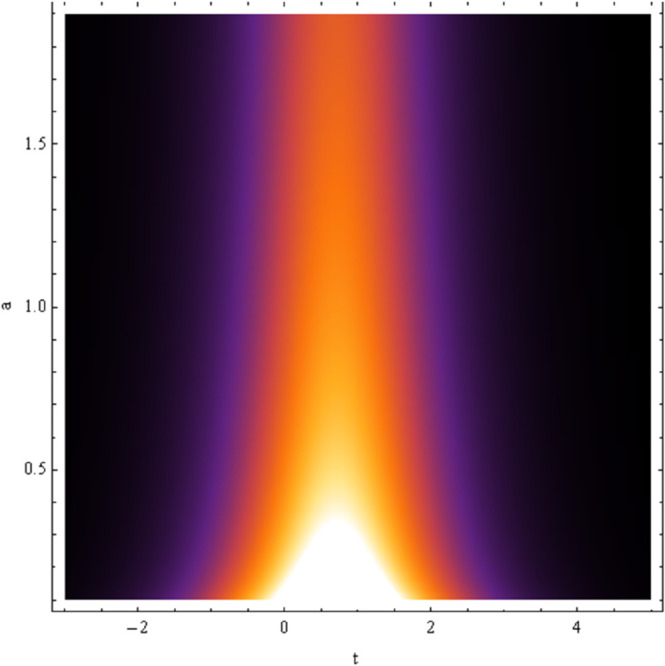
Plot of solution (26) with *a*,*t.*

In absence of noise effect i.e., σ=0, the solitonic solution for equation (26) is shown in Fig 8. The parametric regime for which the soliton wave may be exist depends on the model parameters *a* and ω are shown in Figs 9, 10.

## 5 Conclusions

This study presents a wide range of analytical stochastic solutions for the model of chiral nonlinear Schrödinger problem induced by multiplicative Itô noise. For this purpose, we applied the robust technique in a unified way. Understanding certain significant and intricate physical events requires the use of such solutions. It has been investigated how the noise strength term affects structural characteristics. The resulting wave is modulated and fluctuated by the noise stochastic parameter, resulting in collapsing solitonic tails. Furthermore, the suggested method is a simple and credible tool that can produce significant findings when used with other complex models in the applied sciences.
